# Which Environmental Factors Predict Seasonal Variation in the Coral Health of *Acropora digitifera* and *Acropora spicifera* at Ningaloo Reef?

**DOI:** 10.1371/journal.pone.0060830

**Published:** 2013-04-23

**Authors:** Saskia Hinrichs, Nicole L. Patten, Ming Feng, Daniel Strickland, Anya M. Waite

**Affiliations:** School of Environmental Systems Engineering and The Oceans Institute M470, University of Western Australia, Perth,Western Australia, Australia; Universidade Federal do Rio de Janeiro, Brazil

## Abstract

The impact of physico-chemical factors on percent coral cover and coral health was examined on a spatial basis for two dominant *Acropora* species, *A. digitifera* and *A. spicifera,* at Ningaloo Reef (north-western Australia) in the southeast Indian Ocean. Coral health was investigated by measuring metabolic indices (RNA/DNA ratio and protein concentration), energy levels (lipid ratio) and autotrophic indices (chlorophyll *a* (chl *a*) and zooxanthellae density) at six stations during typical seasons (austral autumn 2010 (March and April), austral winter 2010 (August)) and during an extreme La Niña event in summer 2011 (February). These indices were correlated with 15 physico-chemical factors (measured immediately following coral sampling) to identify predictors for health indices. Variations in metabolic indices (protein concentration and RNA/DNA ratio) for *A. spicifera* were mainly explained by nitrogen, temperature and zooplankton concentrations under typical conditions, while for *A. digitifera*, light as well as phytoplankton, in particular picoeukaryotes, were important, possibly due to higher energy requirement for lipid synthesis and storage in *A. digitifera*. Optimum metabolic values occurred for both *Acropora* species at 26–28°C when autotrophic indices (chl *a* and zooxanthellae density) were lowest. The extreme temperature during the La Niña event resulted in a shift of feeding modes, with an increased importance of water column plankton concentrations for metabolic rates of *A. digitifera* and light and plankton for *A. spicifera*. Our results suggest that impacts of high sea surface temperatures during extreme events such as La Niña may be mitigated via reduction on metabolic rates in coral host. The high water column plankton concentrations and associated low light levels resulted in a shift towards high symbiont densities, with lower metabolic rates and energy levels than the seasonal norm for the coral host.

## Introduction

Coral reefs are under increasing pressure from climate change and human impacts [1]. Episodes of coral bleaching, driven by elevated sea water temperature are predicted to increase in frequency and severity to cause up to 60% coral mortality globally within the next few decades [2]–[4]. In addition, hydrodynamic stresses in the form of extreme storm and wave events are predicted to occur more frequently in the future resulting in the breakage and destruction of corals reefs worldwide [5]–[7]. Coral species that are more resilient to high temperatures and hydrodynamic stresses might therefore dominate reef communities in the future climate [8], [9].

Ningaloo Reef, the world's largest fringing reef, is situated on the northwest coast of Australia in the southeast Indian Ocean. The physico-chemical marine environment off Ningaloo is strongly influenced by climate variability in the Indo-Pacific Ocean [10]–[12]. It has been predicted that climate-driven sea-level rise will affect the hydrodynamic regime of the reef, including changes in local current speeds and light intensity reaching benthic communities [13]. The hydrodynamics of Ningaloo Reef are also influenced by changes in the frequency and/or intensity of storm events due to climate-related variability, leading to changes in the broad current patterns, thermocline depth and subsequent marine productivity off the Western Australia coast [14]. Such large-scale events would likely result in changes in seawater temperatures over the reef, as well as changes in the concentration of nutrients reaching the reef [15].

Given that seasonal and inter-annual changes in physico-chemical factors have already been observed at Ningaloo Reef, including current speeds [13], [16], light, temperature, nutrient concentrations and plankton concentrations [14], [17], [18], we expect that such physico-chemical changes will drive shifts in coral health indices. Knowing how seasonal changes in coral health are related to physico-chemical factors is critical for understanding how extreme events and climate-driven environmental changes will affect coral communities.

For corals to remain healthy in unfavourable conditions, they require sufficient metabolic energy to support growth and reproduction [19], [20] through autotrophic (light-driven) and/or heterotrophic (active uptake of nutrients) processes [21]–[23]. Autotrophic processes depend mainly on the absorption of light and the uptake of dissolved nutrients and result in translocation of photosynthetically fixed carbon by intracellular symbionts (zooxanthellae) to their coral host [22], [24]. In contrast, heterotrophic processes rely on the uptake and subsequent utilisation of plankton, particulate nutrients and dissolved organic matter by corals to support coral host and symbiont metabolic processes [25]–[27]. Whether corals predominantly receive energy through autotrophic or heterotrophic processes has been shown to be species-specific and dependent on seasonally driven changes in the physico-chemical seawater environment [25], [28], [29]. For example, RNA/DNA ratio, protein content, indicators of metabolic activiy, and lipid ratio, an indicator of stored energy, exhibit changes in response to shifts in seawater temperature [30]–[32], light [33], [34], dissolved nutrients [35] and particulate nutrient availabilibity [30], [36], [37]. In addition, shifts in the seawater hydrodynamics can impact both autotrophy and heterotrophy [38]–[40]. For example, changing current speeds can alter the concentration of dissolved and particulate nutrients reaching coral reefs, the boundary layer thickness of individual corals and subsequent coral uptake rates [39], [41]. However, it is not known which physico-chemical factors are the main driving forces for coral metabolism and energy stores, or how they are related to autotrophic indices. To date, no study has determined how changes in the physico-chemical environment may affect a variety of coral health indices simultaneously under normal as well as extreme conditions in the natural environment.

The impacts of seasonal variations as well as extreme events on coral health indices including metabolic activity (RNA/DNA ratio, protein concentration), autotrophic indices (zooxanthellae density, chlorophyll *a* (chl *a* concentration) and energy levels (lipid ratio) were described previously for two dominant *Acropora* species (*A. spicifera* and *A. digitifera*) at Ningaloo Reef [42]. However, it is still unknown which physico-chemical factors were the most important factors driving changes in these coral health indices over seasonal scales and if/how coral health indices further shift during extreme events.

The overall objective of this study was to determine to what extent coral health indices are driven by temporal variability in the physico-chemical environment. To do this we follow up on a previous study [42] and determine the driving physico-chemical factors for predicting coral metabolic activity (RNA/DNA ratio and protein concentration), autotrophic indices (zooxanthellae density and Chl *a* concentration) and energy level (lipid ratio) under normal conditions as well as under extreme weather conditions (La Niña) at Ningaloo reef.

## Methods

### Study site

**Figure 1:**
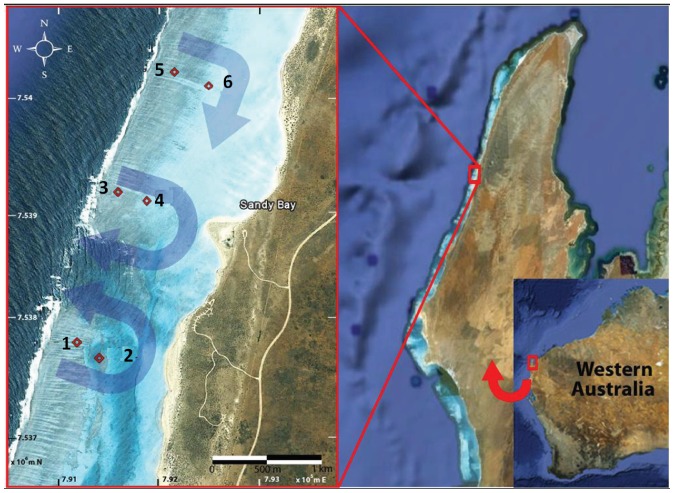
Map of study region and sampling stations (depicted by red diamonds and numbers). Superimposed blue arrows show the characteristic flow pattern across the reef flat and around the lagoon (as described by Lowe et al. 2008). Satellite imagery: Google Earth, 2010.

The study site, Sandy Bay Lagoon (Sandy Bay), is located at Ningaloo Reef, along the North-West Cape of Western Australia (22.23°S, 113.84°E). Sandy Bay lagoon was chosen as hydrodynamic patterns have been described previously [16], [43] and the fringing reef is typical of the ∼290 km stretch of Ningaloo Reef, with shore-parallel reef sections periodically interrupted by channels [16]. The steep reef front (∼1∶50) rises to a shallow reef crest (∼1.5 m), where waves break transporting water into the lagoon across the crest and returning back to the ocean through the channels. At Sandy Bay, the reef crest starts ∼50 m from the surf zone, spreads over ∼500 m and reaches 1000 m shoreward, giving way to a sandy lagoon habitat (depth ∼2–3 m) (Fig. 1).

Sampling was conducted at six stations, two stations south (1, 2) and four stations north (3, 4, 5, and 6) of the channel (Fig. 1). Higher average long-term current speeds occur at stations to the south of the channel compared to stations to the north of the channel [13]. Three of these stations were located close to the ocean side of the reef (1, 3, and 5) and three stations were close to the lagoon (2, 4, and 6) (Fig. 1). The reef crest at Sandy Bay is dominated by the coral genus *Acropora* [44]. Permits to conduct fieldwork were given by the Department of Environment and Conservation (Government of Western Australia).

### Sampling

Two different coral species; the plate form, *Acropora digitifera* (Dana, 1846) and the caespito-corombose form, *Acropora spicifera* (Dana, 1846) [45] (n = 6 at each station) were tagged at each of the six stations. Coral samples were collected between 10:00 and 14:00 in autumn covering the period before coral spawning (28–30^th^ March 2010) and after the coral spawning event (15–17^th^ April 2010), in winter (18 – 27^th^ August 2010) and summer (12–15^th^ February 2011). February 2011 was the peak of a strong La Niña event and oceanic conditions were warmer and more nutrient rich than in typical summers in this region [46]. Corals were sampled while snorkelling. Three 1–5 cm long coral pieces were removed from the middle of the coral colony (tip of the branch) for each of the health indices (see below).

### Analysis of health indices

#### Coral cover

Estimates of percent hard coral cover (% coral cover) at each station were determined from photographs taken along two parallel transects, of 100 m length, which were spaced 10 m apart. Photographs were taken at 5 m intervals along the 100 m transect. A metal disc of known diameter was placed in each photograph and the time recorded to allow photo area and the corresponding tide adjusted depth to be calculated from each photograph. Percent hard coral cover was estimated for each coral category (plate corals such as *A. spicifera*, caespito-corymbose corals such as *A. digitifera* as well as all other live hard coral) in each photograph. A pixel counting technique was facilitated in *MATLAB* after visually inspecting and colouring the corals with solid, distinct colours (e.g. red, green, blue) in *Microsoft Paint*. The time each transect was conducted was combined with local tidal records to standardize all estimated depths to a 2.2 m tide.

#### Zooxanthellae density and Chlorophyll a

Coral samples (∼3–5 cm long) for the analysis of zooxanthellae density (per cm^2^) and chl *a* concentration in the tissue (µg cm^−2^) were stored at −20°C prior to analysis. Methods for zooxanthellae density in coral tissue were adapted from Siebeck et al. [47]. Chl *a* concentration was determined following the methods of Jeffrey and Humphrey [48]. Coral surface area was measured using the paraffin wax method [49].

#### RNA/DNA and protein analysis

Samples (1 cm long) for RNA/DNA and protein analysis were stored at −80°C prior to analysis. Methods for analysis of RNA/DNA followed Humphrey [50]. For protein analysis, a standard DC Protein Kit was used [51].

#### Lipid ratio

Coral pieces (∼3 cm long) for lipid ratio analysis were stored at −20°C until freeze-dried for 24 hours in the laboratory. Methods for lipid analysis followed those described by Saunders et al. [52].

### Analysis of physico-chemical-biological factors

Physico-chemical factors known to be important for coral health were sampled immediately following coral sampling. These included light, temperature, current speed, nutrients (nitrogen, phosphate, and silicate), and phytoplankton and zooplankton concentrations.

#### Physical factors

Light, measured as photosynthetically active radiation (PAR), and temperature were measured using Hobo Pendant Data Loggers deployed at each station at the benthos over the sampling period for 2 weeks.

Short-term current speed was estimated using drifters. Two crucifix design drifters [43] containing a GPS were simultaneously deployed for approximately 10 minutes and the GPS location and time of their deployment and collection recorded. The distance and time of each deployment allowed a simple estimate of surface flow speed. Long-term estimates (≈ months) of current speed were based on data from a previous investigation at the site. Data [13], [16] provided average current speed near the reef crest for stations 1, 3 and 5 and were based on hourly current measurements of NORTEK Vector ADVs deployed 0.5 m above the sea floor. Estimates of mean current speed at stations 2, 4 and 6 were derived by applying conservation of mass for flow near the reef crest (station 1, 3, 5), based on the ratio of average depths at the sites (estimated by analysis of photo-quadrat size). The technique provides very conservative estimates of mean current speed, assuming flow is direct between the two sites and that vertical flow profiles at each are comparable.

#### Chemical and biological factors in the water column

Water samples were taken for the analysis of dissolved nutrients (Nitrate + Nitrite (NO_x_), ammonium (NH_4_), phosphate (PO_4_) and silicate (Si)), total nitrogen (TN) chl *a* (chl *a*<2 µm, chl *a*<5 µm and Total chl *a*) and picoplankton (*Synechoccocus*, *Prochloroccocus* and picoeukaryotes). Chl *a*<5 µm is defined as large phytoplankton.

For the analysis of dissolved NO_x_, NH_4_, PO_4_ and Si, water samples (40 ml) were filtered through 0.45 µm filters and stored at −20°C, before flow injection analysis (FIA) with detection by absorbance at specific wavelengths for Si (QuikChem Method 31-114-27-1-D), NO_x_ (Quickchem Method 31-107-04-1-A) and PO_4_ (QuikChem Method 31-115-01-1-G) [53]. NH_4_ was measured by fluorescence (GlobalFIA high sensitivity gas diffusion unit- HPMSD, Shimadzu RF-10Axl Fluorescence detector) [54]. For TN, unfiltered water samples (50 ml) were stored at −20°C until analysis. TN was determined from autoclave digests with potassium persulphate (Lachat Quick-Chem 8500 Automated Flow Injection Analyser) [55].

Seawater samples (1 l) for the Total chl *a* size fraction were filtered onto Whatman GF/F filters (norm size 0.7 µm); seawater samples (0.5 l) for chl *a*<2 µm fraction were filtered onto 2 µm nuclepore polycarbonate membrane filters and seawater samples (2 l) for chl *a*<5 µm fraction were filtered onto 5 µm nitex mesh. The filters were stored at −20°C in the dark until fluorometric analysis of duplicate 90% acetone extracts was conducted [56]. Chl *a*<2 µm fraction was calculated by subtracting chl *a*>2 µm from Total chl *a* and chl *a* 2<5 µm was calculated by subtracting chl *a*>5 µm from chl *a*>2 µm.

To determine concentrations of autotrophic picoplankton groups (*Synechoccocus*, *Prochlorococcus* and picoeukaryotes), duplicate 1.5 ml seawater samples were fixed with gluteraldehyde (0.5% final concentration) for 10 minutes and then snap frozen in liquid nitrogen. Samples were analysed using flow cytometry [17]; using a FACSCANTO II (Becton-Dickinson) flow cytometer fitted with a 488 nm laser on high throughput mode at a flow rate of 60 µl min^−1^ for 100 s.

Zooplankton were sampled with a 90 µm plankton net towed for 10 min at 2.5 kn h^−1^ for 50 m at each station and preserved with formaldehyde (ca. 5%). Dry weight analyses were carried out for zooplankton size fractions of 100 to 500 µm and 500 to 1000 µm. Containers were weighted, wet samples added after cleaning samples of salt with deionised water (30 s) and dried in an oven (60°C) for 24 hours. Afterward samples were cooled in a desiccator and weighted for subsequent calculation of dry weight (DW) [57].

### Statistical analysis

Correlations between five coral health indices (RNA/DNA ratio, protein concentration, lipid ratio, chl *a* concentration and zooxanthellae density) and 15 physico-chemical factors (light, temperature, current speed (drifter), water column chl *a* concentration (< 2 µm, 2–5 µm, >5 µm), picoplankton (*Synechoccocus*, *Prochlorochoccus*, picoeukaryotes), zooplankton dry weight (100–500 µm and 500–1000 µm) and dissolved nutrients (NO_x_, NH_4_, Si, PO_4_)) were tested with DISTLM (Primer 6) based on Bayesian information criterion (BIC) (forward procedure, 9999 permutations) [58]. Data for health indices were log-transformed based on results of draftmans plot and Grubb's test to reduce outliers and make data more continuous. Physico-chemical data were square root transformed and DISTLM was performed based on Euclidean dissimilarity matrix. This test was done 1) for all data excluding the La Niña event as well as 2) only including the spawning event (autumn) and 3) all data including the La Niña event (in this case data were square root transformed except chl a concentration (log-transformed) and zooplankton concentrations (forth root transformed) to determine which physico-chemical factors drive coral health in the presence and absence of extreme events. Seasonal differences in physico-chemical factors were tested with PERMANOVA after transformation based on Euclidean distance using 9999 permutations with Type III (partial) sum of square and permutation of unrestricted permutation of raw data [58]. In addition, univariate relationships were determined for PAR and temperature with each health indices since temperature exhibited quadratic function with all health indices (except lipid ratios of A. spicifera) (Sigma Plot). To determine correlations between % coral cover and long-term current speed the coefficient of determination R2 was tested with DISTLM (Primer 6) based on Euclidian dissimilarity matrix (9999 permutations, with selection criteria all specified) [58].

## Results

### Relationships between coral cover and current speed

**Figure 2:**
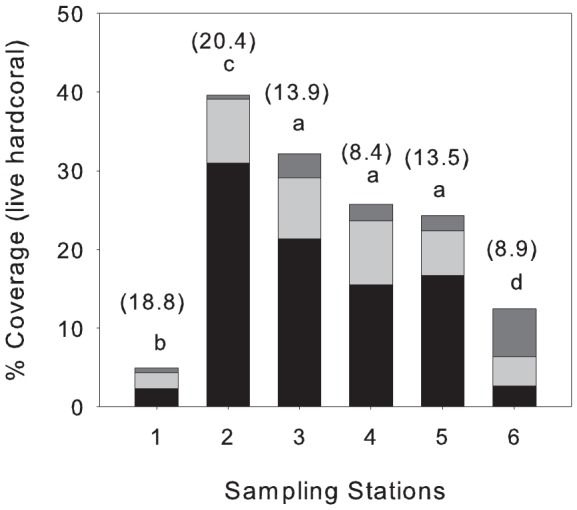
Percent coral coverage of plate *Acropora*, caespito-corymbose *Acropora* and other coral species at Ningaloo reef. Six stations were sampled in autumn (March - April 2010). Numbers in brackets represent long-term average current speed per station and same letters indicate no significant difference in total coral coverage per station.

The mean % coral cover of *A. spicifera*, *A. digitifera* and other live hard corals at the six stations at Ningaloo Reef are displayed in Figure 2. Total % coral cover ranged between 4.9% and 39.6% across the six stations, with lowest and highest % total coral cover occurring at station 1 and station 2 respectively and with similar coral cover occurring at station 3, 4 and 5. *A. spicifera* dominated coral cover at each of the stations, averaging 56.9% of the total % coral cover (ranging between 21.1 and 78.3%). At station 1, *A. spicifera* and *A. digitifera* displayed similar coverage (ca. 2%), disproportionate to other near-reef crest sites (station 3 and 5) where *A. spicifera* displayed ca. 3-fold greater coverage then *A. digitifera* (Fig.2).

Long-term averaged current speeds ranged between 8.4 and 20.3 cm s^−1^, with highest and lowest current speeds occurring at station 2 and 4 respectively (Fig. 2). Overall, % coral cover was not correlated with long-term current speed (p>0.1). However, the relationships were confounded by comparatively low coral cover occurring at station 1, corresponding with high average (long-term) flow speed but also due to a high density of *Drupella* spp. found at this station. The removal of this site from the data set improved the correlations considerably with high R^2^ values for relationship between long-term current speed and plate *Acropora* (R^2^ = 0.76) and total % coral cover (R^2^ = 0.69), however correlations were still not significant probably due to small sample size (n = 5).

### Seasonal changes in physico-chemical factors

**Figure 3:**
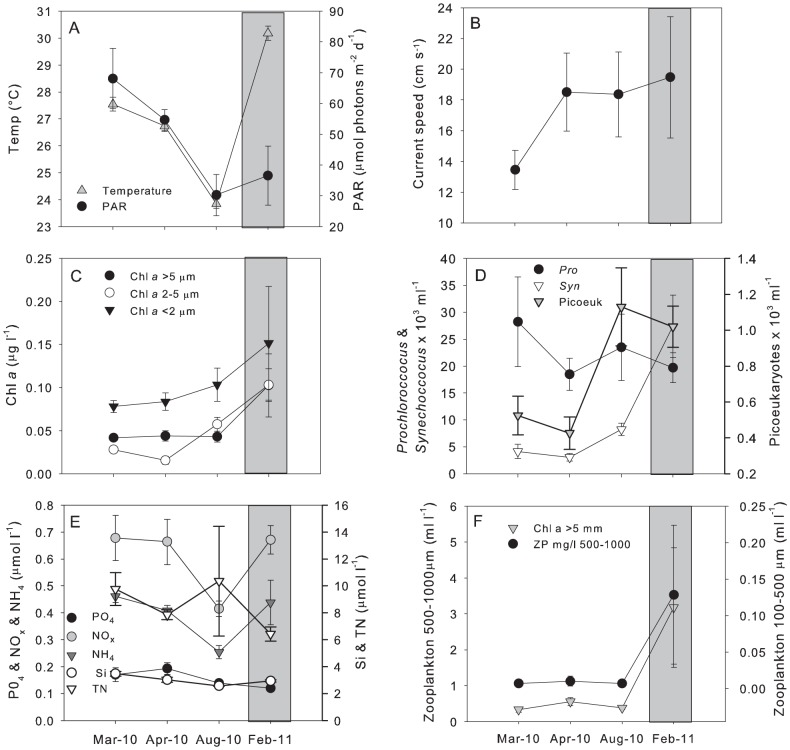
Patterns in physico-chemical factors at Ningaloo Reef throughout seasons. (A) Temperature (°C) and light (PAR), (B) current speed, (C) chlorophyll *a* (chl *a*), (D) picoplankton concentration (*Prochloroccocus*, *Synechoccocus* and picoeukaryotes ), (E) nutrients (PO_4_, NO_3_+NO_2_ (NO_x_), NH_4_, Si and TN) and (F) zooplankton concentrations at six stations at Ningaloo Reef during autumn (March/April) and winter (August 2010) and during the La Niña event in summer (February 2011) (grey column). Data are means ± SE (n = 6).

PAR and temperature both decreased from autumn (March/April 2010) to winter (August 2010) from 70 to 30 µmol m^−2^ d^−1^ and 27 to 23°C respectively, while current speed increased from 14 to 18 cm s^−1^. NO_x_ as well as NH_4_ showed a decrease from autumn to winter while TN as well as water column chl *a*, *Synechoccocus* and picoeukaryotes showed increasing concentrations (Fig. 3). *Prochlorochoccus* dropped from 30 × 10^6^ to 20 × 10^6^ cells ml^−1^ from autumn to winter, while zooplankton concentration did not significantly vary between seasons.

During the study, a near-record La Niña event occurred during the austral summer of 2010-2011 (sampling period in February 2011). At this time, seawater temperature reached a maximum of 30.8°C, while current speed and PAR were variable, ranging between 5.8 and 31.5 cm s^−1^ and between 14.8 and 70.2 µmol photons m^−2^ d^−1^ respectively, with these values most comparable to the winter situation in August 2010 (Fig. 3). Significant differences for temperature occurred between summer (February 2011) and autumn 2010 and summer 2011 and winter 2010 (F_3, 23_ = 134.3, P<0.001, pair-wise comparison P<0.05) and for PAR between summer 2011 and autumn 2010 (F_3, 23_ = 4.61, P<0.05, pair-wise comparison P<0.05).

Water column chl *a* (all size fractions) peaked during the La Niña event, with concentrations two times higher than during any other sampling period, reaching a maximum of 0.10 µg l^−1^ for both chl *a* 2 – 5 µm and chl *a*>5 µm, and 0.15 µg l^−1^ for chl *a*<2 µm. However, only chl *a* in the 2–5 µm size fraction differed significantly to autumn 2010 and winter 2010 (F_3, 23_ = 13.9, P<0.001, pair-wise comparison p<0.05). The trend for picoplankton was more complex and varied for each group. *Synechococcus* concentrations were five times higher in summer 2011 than during any other season (F_3, 23_ = 19.03, P<0.001, pair-wise comparison P<0.05). Picoeukaryote concentrations peaked in winter 2010 at 1.13 × 10^6^ cells ml^−1^, however were comparably high in summer 2011 (F_3, 23_ = 7.01, pair-wise comparison P>0.05), while *Prochloroccocus* concentrations were lowest during the summer La Niña event but did not show significant seasonal differences (P>0.05). The same trend as observed for chl *a* concentration and *Synechococcus* occurred for zooplankton concentrations three times higher during the La Niña summer. Highest zooplankton concentrations occurred in the 500 – 1000 µm fraction in summer 2011 and differed significantly to those in autumn and winter 2010 (F_3, 22_ = 3.56, P<0.05, pair-wise comparison p<0.05). Zooplankton in the 100 – 500 µm size fraction also showed highest values in summer however significant differences only occurred between summer 2011 and autumn 2010 (F 3,23 = 3.49, P<0.05, pair-wise comparison P>0.05) (Fig. 3).

Nutrient concentrations during the summer La Niña event were similar to autumn 2010, with relatively high NO_x_ as well as NH_4_ concentrations that differed significantly from concentrations in winter 2010 (NO_x_: F_3, 23_ = 5.16, P<0.01; NH_4_: F_3, 23_ = 5.25, P<0.01; pair-wise comparison P<0.05). Lower concentrations of PO_4_ were observed in summer 2011 compared to autumn and winter 2010 (F_3, 23_ = 4.12, P<0.05; pair-wise comparison P<0.05) (Fig. 2). TN showed relatively low values in summer 2011 compared to those in autumn 2010 with significantly different values from March 2010 (F_3, 22_ = 7.17, P<0.001; pair-wise comparison P<0.05) (Fig. 3).

### Relationships between coral health indices and physico-chemical factors

To determine which physico-chemical factors were most important for metabolic activity, autotrophic indices and energy stores for *A. digitifera* and *A. spicifera,* multivariate analysis was performed across seasons that showed 1) typical seasonal patterns in physico-chemical factors (autumn 2010 and winter 2010); 2) during autumn 2010 alone (during the spawning event), since metabolic rates were highest during this time [42] and 3) during all sampled seasons including the La Niña event (Autumn 2010, Winter 2010 and Summer 2011 La Nina) (Table 1, 2). Relationships between physico-chemical factors with each measured coral health indices are described below.

**Table 1:** Main physico-chemical predictors for coral health indices for *Acropora digitifera*.

	Typical seasons (Autumn and Winter 2010)				Spawning event (Autumn 2010)				All seasons (Autumn & Winter 2010 and Summer 2011)			
Health indices	Predictor	BIC	Pseudo-F	% var	Predictor	BIC	Pseudo-F	% var	Predictor	BIC	Pseudo-F	% var
**Protein**	**Chl ** ***a*** 2–5 µm (µg l^−1^) ***	−292.9	103.48	54.9	**Chl ** ***a*** 2–5 µm (µg l^−1^) ***	−229.7	18.35	23.1	**Chl ** ***a*** ** 2–5** µm (µg l^−1^) ***	−357.6	106.67	49.5
**(mg µg^−1^DNA)**	Temp (°C) ***	−312.8	27.15	11,00	Temp (°C) *	−231.7	6.09	7.1	ZP 100–500 µm (mg l^−1^) ***	−382.9	33.53	12,00
	PO_4_ (µmol l^−1^) ***	−319.7	11.58	4.2	NO_3_ + NO_2_ (µmol l^−1^)*	−231.8	4.11	4.5	Si (µmoll^−1^) ***	−395.4	17.87	5.5
	Pro (cells µl^−1^) *	−320.1	4.72	1.6	N/A	N/A	N/A	N/A	N/A	N/A	N/A	N/A
**RNA/DNA**	PAR (µE m^−2^ s^−1^) ***	−213.8	39.06	31.5	PAR (µE m^−2^ s^−1^) **	−171.7	7.43	10.9	**Chl ** ***a*** ** 2–5** µm (µg l^−1^) ***	−257.9	55.48	33.7
	Chl *a*>5 µm (µg l^−1^) *	−216,00	6.68	5.1	N/A	N/A	N/A	N/A	Pro (cells µl^−1^) ***	−277.3	26.23	13,00
	N/A	N/A	N/A	N/A	N/A	N/A	N/A	N/A	Picoeuk (cells µl^−1^) **	−280.6	8,00	3.7
**Lipid ratio**	Picoeuk (cells µl^−1^) ***	−362.9	27.82	24.7	Pro (cells µl^−1^) *	−255.2	6.61	9.8	Picoeuk (cells µl^−1^) ***	−465.9	28.19	20.5
	Si (µmol l^−1^) **	−370.1	12.05	9.5	Si (µmol l^−1^) **	−258.5	7.5	10,00	Si (µmol l^−1^) ***	−475.4	14.74	9.5
**Chl ** ***a*** ** (µg cm^−2^)**	Picoeuk (cells µl^−1^) ***	^−^191.3	78.6	48,00	Chl *a*<2 µm (µg l^−1^) ***	−164.9	35.57	36.8	Picoeuk (cells µl^−1^) ***	−241.2	94.9	46.5
	Temp (°C) ***	−213.8	30.56	13.9	**Chl ** ***a*** **>5 µm (µg l** ^−**1**^ ) *	−167.3	6.64	6.3	**TN (µmol l** ^−**1**^ ) ***	−260,00	25.48	10.2
	PAR (µE m^−2^ s^−1^) **	−219.5	10.3	4.2	N/A	N/A	N/A	N/A	PAR (µE m^−2^ s^−1^) **	−266.3	11.21	4.1
**ZD (cells cm^−2^)**	Temp (°C) ***	−182.2	36.53	30.1	Temp (°C) *	−138.4	4.52	6.9	PAR (µE m^−2^ s^−1^) ***	−230.9	32.8	23.1
	Picoeuk (cells µl^−1^) *	−182.8	5.07	4,00	N/A	N/A	N/A	N/A	NH_4_ (µmol l^−1^) **	−236.3	10.29	6.7
	N/A	N/A	N/A	N/A	N/A	N/A	N/A	N/A	Picoeuk (cells µl^−1^) *	−237.7	6.04	3.8

Typical seasons (autumn and winter 2010) are not affected by La Niña event, during the coral spawning event in autumn 2010 metabolic indices were highest and when all sampled seasons are tested also the La Niña event is included (autumn 2010, winter 2010 and summer 2011). Tests were performed with DISTLM based on BIC (Primer 6). Bold letters indicate significant negative relationships and non bold ones significant positive relationships.* P<0.05; ** P<0.01; *** P<0.001. Var  =  variation, Pro  =  *Prochloroccocus*, Picoeuk  =  picoeukaryotes, Temp  =  temperature, ZP  =  zooplankton, TN  =  total nitrogen.

**Table 2:** Main physico-chemical predictors for coral health indices for *Acropora spicifera*.

	Typical seasons (Autumn and Winter 2010)				Spawning event (Autumn 2010)				All seasons (Autumn & Winter 2010 and Summer 2011)			
Health indices	Predictor	BIC	Pseudo-F	% var	Predictor	BIC	Pseudo-F	% var	Predictor	BIC	Pseudo-F	% var
**Protein**	Temp (°C) ***	−100.8	44.32	35.7	NH_4_ (µmol l^−1^) *	−50	18.35	8.8	Picoeuk (cells µl^−1^) ***	−128	33.51	24.4
**(mg µg^−1^DNA)**	NH_4_ (µmol l^−1^) *	−102.3	5.89	4.5	Current speed (cm s^−1^)*	−50.1	6.09	6.6	PAR (µE m^−2^ s^−1^) ***	−140.8	18.49	11.5
	ZP 500–1000 µm (mg l^−1^) *	−104.5	6.54	4.6	N/A	N/A	N/A	N/A	PO_4_ (µmol l^−1^) **	−144.4	8.26	4.8
**RNA/DNA**	Temp (°C) ***	−108.5	13.39	14.3	ZP 500–1000 µm (mg l^−1^)	−75.5	4.11	4.7l	**Chl ** ***a*** 2–5 µm (µg l^−1^) ***	−142.5	14.94	12.6
	N/A	N/A	N/A	N/A	N/A	N/A	N/A	N/A	PAR (µE m^−2^ s^−1^) *	−144.3	6.42	5.1
**Lipid ratio**	TN (µmol l^−1^) *	−332.7	5.52	6.5	TN (µmol l^−1^) *	−223.1	7.43	11.7	TN (µmol l^−1^) *	−421.4	4.31	4
	Si (µmol l^−1^) **	−338.4	10.39	10.9	Si (µmol l^−1^) **	−228.1	6.61	13.3	Si (µmol l^−1^) **	−424.6	7.91	6.8
**Chl ** ***a*** ** (µg cm^−2^)**	Temp (°C) **	−182.7	52.51	39.6	Chl *a* 2–5 µm (µg l^−1^) **	−139.9	7.5	18.5	Syn (cells µl^−1^) ***	−193.9	59.73	36.5
	Picoeuk (cells µl^−1^) *	−185.5	07.31	5.1	NH_4_ (µmol l^−1^) **	−145.7	35.57	13.3	PAR (µE m^−2^ s^−1^) ***	−214.1	27.13	13.2
	ZP 500–1000 µm (mg l^−1^) *	−187	05.83	3.8	N/A	N/A	N/A	N/A	**Chl ** ***a*** >5 µm (µg l^−1^) ***	−222.8	13.73	6
	TN (µmol l^−1^) *	−187.8	5.04	3.2	N/A	N/A	N/A	N/A	ZP 500–1000 µm (mg l^−1^) *	−222.9	4.67	2
**ZD (cells cm^−2^)**	NH_4_ (µmol l^−1^) *	−178.8	6.68	7.7	NO_3_ + NO_2_ (µmol l^−1^)*	−156.7	6.64	9.1	PAR (µE m^−2^ s^−1^) **	−213.1	11.56	10

Typical seasons (autumn and winter 2010) are not affected by La Niña event, during the coral spawning event in autumn 2010 metabolic indices were highest and when all sampled seasons are tested also the La Niña event is included (autumn 2010, winter 2010 and summer 2011). Tests were performed with DISTLM based on BIC (Primer 6). Bold letters indicate significant negative relationships and non bold ones significant positive relationships.* P<0.05; ** P<0.01; *** P<0.001. Pro  =  *Prochloroccocus*, Syn  =  *Synechoccocus*, Picoeuk  =  picoeukaryotes, Temp  =  temperature, ZP  =  zooplankton, TN  =  total nitrogen.

In general, picoplankton and chl *a* concentration in the water column were negatively correlated with protein concentration and lipid ratio, while temperature, PAR, dissolved nutrients (with exception of TN) and zooplankton were positively correlated with protein concentration and lipid ratio for both *Acropora* species. In contrast, chl *a* in coral tissue and zooxanthellae density exhibited negative correlations with temperature, PAR and dissolved nutrients but positive correlations with plankton (picoplankton, chl *a* and zooplankton with the exception of water column chl *a*>5 µm) for both *Acropora* species. Correlations between RNA/DNA ratio and PAR, temperature chl *a*>5 µm and zooplankton were positive (Table 1, 2).

#### During typical seasonal patterns (autumn and winter 2010)

For *A. digitifera*, water column chl *a* in the 2–5 µm size fraction explained 54.9% of the protein concentration variability. Temperature, PO_4_ and *Prochlorococcus* explained a further 16.8% of the variability in protein concentration (Table 1). In contrast for *A. spicifera*, 44.7% of the variability in protein concentration was best explained by temperature (35.7%), NH_4_ (4.5%) concentration and zooplankton in the 500–1000 µm size fraction (4.6%) (Table 2).

For *A. digitifera*, RNA/DNA ratios exhibited strongest correlations with PAR (31.5% of the variability) and to a lesser extent, with chl *a*>5 µm (5.1% of the variability) (Table 1). Temperature best explained RNA/DNA ratio variability for *A. spicifera* (14.3%) (Table 2).

For both *A. digitifera* and *A. spicifera*, lipid ratios were correlated with Si concentrations in the water column, explaining 9.5% and 10.9% of the variability respectively. For *A. digitifera*, 24.7% of the variability in lipid ratios was best explained by picoeukaryote, while for *A. spicifera* 6.5% of protein concentration variability was explained by TN (Table 1, 2).

Picoeukaryote concentration in the water column was responsible for 48% of the variability of tissue chl *a* for *A. digitifera*, with temperature and PAR then explaining a further 18.1% of this variability (Table 1). For *A. spicifera*, temperature showed the highest correlation with tissue chl *a*, explaining 39.6% of the variance, with picoeukaryotes and zooplankton in the 500–1000 µm fraction and TN explaining a further 12.1% of the variability (Table 2).

For A.*digitifera*, temperature explained 30.1% of the variation in zooxanthellae density followed by picoeukaryotes (4.0%) (Table 1), while zooxanthellae density in *A. spicifera* was most strongly correlated with NH_4_ (7.7%) (Table 2).

#### During the coral spawning event (autumn 2010)

During autumn 2010 over the spawning period when metabolic activity was at its maximum, water column chl *a* in the 2–5 µm size fraction was still the best predictor of protein concentration for *A. digitifera* (23.1% of the variability), with temperature and NO_x_ explaining a further 11.6% (Table 1). At this same time for *A. spicifera*, NH_4_ was the main predictor of protein concentration (8.8%) followed by current speed (6.6%) (Table 2).

PAR remained the best predictor of RNA/DNA ratio for *A. digitifera* (10.9%) during autumn (Table 1), while for *A. spicifera* no significant correlation was found with any of the measured physico-chemical factors (Table 2).

For lipid ratios, the main predictors remained the same as during typical seasons. However for *A. digitifera*, *Prochloroccocus* instead of picoeukaryotes showed the strongest correlation with lipid ratio (Table 1, 2).

Water column chl *a*<2 µm was the main predictor of tissue chl *a* for *A.digitifera*, explaining 36.8% of the variability, while water column chl *a*>5 µm explained a further 6.3% of the variability (Table 1). For *A. spicifera*, water column chl *a* in the size fractions 2 – 5 µm exhibited the strongest correlation with tissue chl *a* (18.5%) followed by NH_4_ (13.3%) (Table 2).

During autumn 2010, temperatures remained the best predictor of zooxantheallae density in *A. digitifera* while NO_x_ were responsible for 9.1% in the variation in zooxanthellae density in *A. spicifera* (Table 1, 2).

#### During all sampled seasons including the summer La Nina event (autumn and winter 2010 and summer 2011)

During the La Niña event, the physico-chemical water column environment was very different to that during typical summers [46]. These differences in the physico-chemical environment had a significant impact on the measured coral health indices, with overall lower metabolic rate indicators and lipid stores and higher zooxanthellae densities and chl *a* concentrations in the tissue during the La Niña event then expected during typical summer [42].

For *A. digitifera*, chl *a* in the 2–5 µm size fraction was the best predictor of protein concentration, explaining 49.5% of variability. Zooplankton in the 100–500 size fraction and Si explained a further 17.5% of the variability (Table 1). For *A. spicifera* variability in protein concentration was best predicted by picoeukaryote concentration (24.4%) followed by PAR (11.5%) and PO_4_ concentration (4.8%) (Table 2).

RNA/DNA ratio of *A. digitifera* showed the strongest correlations with water column chl *a* in the 2–5 µm size fraction, explaining 33.7%, with *Prochloroccocus* and picoeukaryotes the next best predictors of RNA/DNA ratio (Table 1). RNA/DNA ratios of *A. spicifera* were correlated with water column chl *a* in the 2–5 µm size fraction and PAR explaining 12.6 and 5.1% of the variability, respectively (Table 2).

Variation in lipid ratio for both *Acropora* species was still correlated with those same physico-chemical factors whether statistical analyses were executed with or without inclusion of the La Niña event. (Table 1, 2)

Picoeukaryotes and PAR were still predictors for chl *a* concentration in the tissue of *A. digitifera* with 46.5% and 4.1%, respectively. Another 10.2% of the variability of chl *a* was explained by TN (Table 1). Variability of chl *a* in the tissue of *A. spicifera* was most strongly correlated with *Synechoccocus* (36.5%). PAR, large phytoplankton and zooplankton in the 500–1000 µm size fraction explained another 21.2% (Table 2).

For variability in zooxanthellae density, PAR and NH_4_ explained 23.1 and 6.7% respectively for *A. digitifera* and picoeukaryotes explain further 3.8% (Table 1). Variability of zooxanthellae density for *A. spicifera* showed strongest correlation with PAR, explaining 10.0% of the variability (Table 2).

### Importance of PAR and nutrients for coral health indices

**Figure 4:**
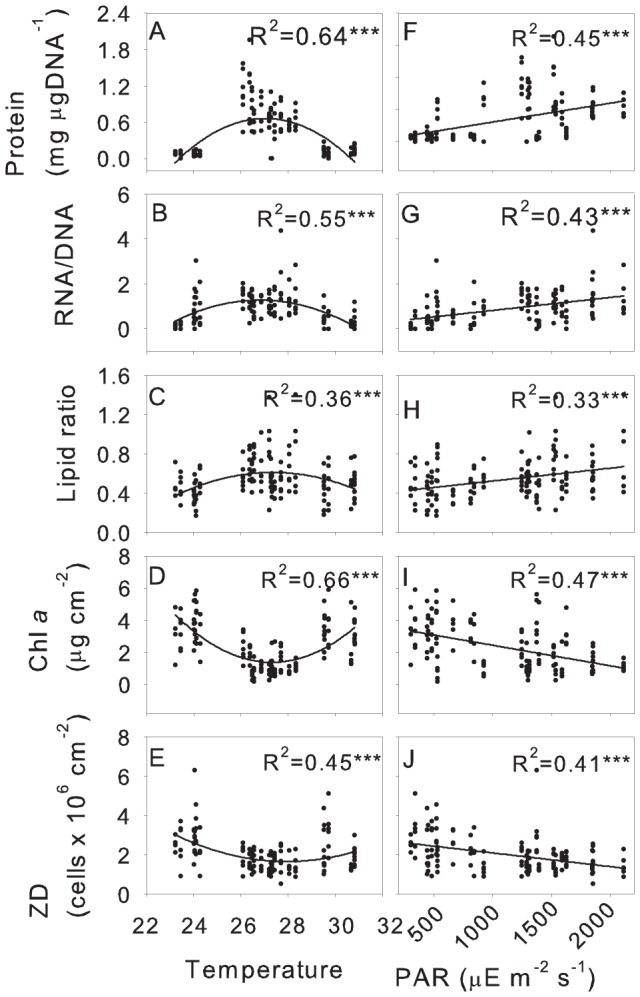
Correlation between health indices with temperature and light for *Acropora digitifera.* Quadratic correlations (R^2^) of temperature (A–E) and linear correlations (R^2^) of PAR (F–J) with Protein concentration, RNA/DNA ratio, lipid ratio, chl *a* concentration and zooxanthellae density.

**Figure 5:**
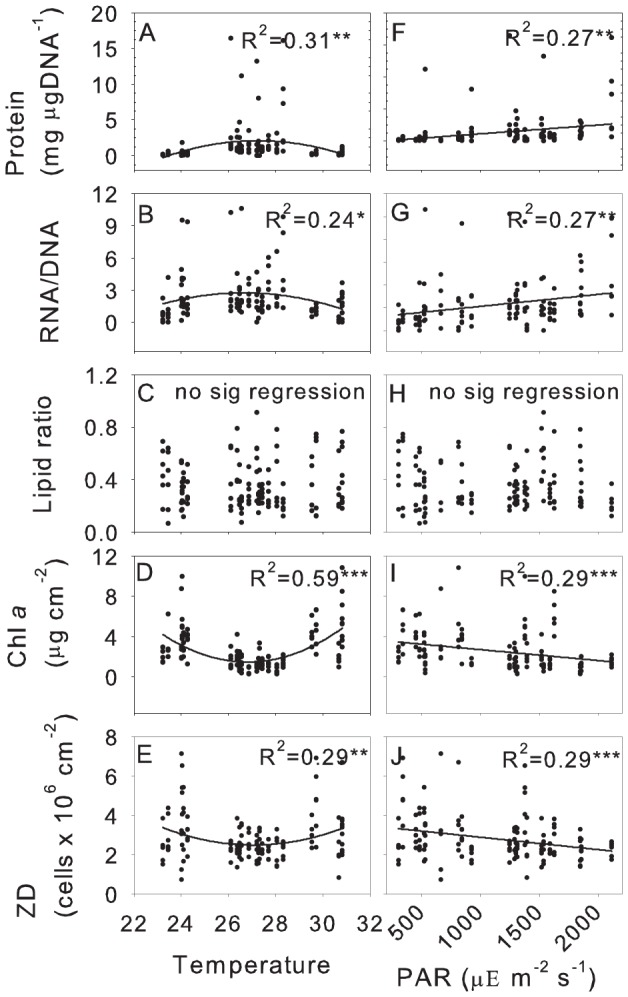
Correlation between health indices with temperature and light for *Acropora spicifera.* Quadratic correlations (R^2^) of temperature (A–E) and linear correlations (R^2^) of PAR (F–J) with Protein concentration, RNA/DNA ratio, lipid ratio, chl *a* concentration and zooxanthellae density.

Since our results suggested that PAR and temperature were important drivers of the variability in the measured coral health indices (Table 1), univariate relationships between these factors were further tested (Fig. 4, 5). Temperature displayed a non-linear relationship with all health indices (except lipids in the case of *A. spicifera*), resulting in highest metabolic rates and lowest autotrophic indices occurring between 26 and 28°C for both *Acropora* species(Fig. 4, 5). Temperatures above or below 26 and 28°C led to decreased metabolic rates but increased autotrophic indices. Metabolic values increased linearly with increasing PAR intensity while autotrophic values declined for both *A. spicifera* and *A. digitifera* (Fig. 4, 5). Linear relationships between lipid ratios and temperature and PAR showed the same trend as metabolic indices for *A. digitifera* with a linear increase with increasing PAR intensity (Fig. 4), however there was no significant relationship between lipid ratios and PAR or temperature for *A. spicifera* (Fig. 5).

## Discussion

Here we show that under natural environmental conditions, there are clear species differences between the two dominant *Acropora* species at Ningaloo Reef, Western Australia, in their physiological response to physical, chemical and biological variables in the marine environment. We argue that *A. digitifera* is more dependent than *A. spicifera* on autotrophy, and uses autotrophy to develop long-term energy stores as lipids. In contrast, *A spicifera* has higher metabolic rates overall, depends more on heterotrophic food sources, and shows the greatest vulnerability to physical stressors at high current speeds. It follows that changes in environmental conditions, including climatic shifts, are likely to impact the community quickly, as demonstrated through our documentation of major changes in coral health through a natural La Niña event.

### Long-term effect of current speed on *Acropora* species coral cover

Results of % coral cover suggests that averaged long-term current speed plays a role in determining reef community composition spatially at Ningaloo Reef. The high % coral cover of plate shaped corals including the dominant plate form *A. spicifera* occurred at sites with high averaged long-term current speed while for caespito-corymbose shaped corals such as *A. digitifera*, no difference in % coral cover was found. Current speeds shape boundary layer dynamics around corals and subsequently affect the rate at which corals can take up dissolved and particulate nutrients from the water column [39], [41]. Since protein concentrations in *A. spicifera* during autumn, when temperatures were optimal, was correlated with current speed and NH_4_, current speed may play a role on the long-term health of corals. However plate shaped corals appear more impacted by acute physical stress events such as cyclones and storm surges [9] than caespito-corymbose corals. This might explain the disproportionate coverage of plate *Acropora* at station 1 which was/is likely to be exposed to these events [59]. Alternatively, shifts in coral-algal dynamics can also result in decreased coral cover. For example algal overgrowth of corals can lead to hypoxia, increased pathogens and viruses along the coral-algal interface [60], [61] and thickening of the boundary layer around corals [62]. Shifts from coral to algal dominated systems are linked with anthropogenic influences including increased nutrients in the water column e.g. from land run off [60] and overfishing [61]. However, at Ningaloo, we do not expect algae coverage to be the main driver of changes in coral health since this area is not particularly exposed to anthropogenic impacts.

### Physico-chemical predictors for coral health during typical seasonal patterns (excluding the La Niña event)

#### A. digitifera

*For A. digitifera*, PAR and temperature were the main predictors for both autotrophic and metabolic indices, suggesting that this species relies to a large degree on photosynthetically derived products. The statistical decline of chl *a* and zooxanthellae density with high PAR and temperatures is likely to be the result of photoacclimatisation [63]. Zooxanthellae densities can vary between 0.5 × 10^6^ to 5 × 10^6^ cells per cm^2^ over time [64], [65] in response to seasonal variability such as PAR and temperature [66], [67]. Despite lower zooxanthellae densities under high PAR conditions, previous studies have shown that photosynthetic rates are higher than under low PAR conditions resulting in an increase of photosynthetically derived products to the coral host [68]. The positive correlation of PAR with RNA/DNA ratio could thus be related to increased translocation of photosynthates to the coral host under high PAR conditions resulting in increased metabolic activity [22], [69]. Previous studies have indeed shown an increase of RNA/DNA ratios with PAR intensity [33], [49], [70] and negative correlations between autotrophic indices and RNA/DNA ratio [42].

Results of this study also reveal that picoplankton, particularly the flow cytometrically identified picoeukaryote group, are possibly important predictors for coral metabolic activity, autotrophic indices and energy stores in *A. digitifera*. This implies that *A. digitifera* relies also on heterotrophy in addition to autotrophy, with heterotrophy possibly providing an additional nitrogen source. Photosynthates translocated to the coral host do not provide corals with sufficient nitrogen [23], [71] and picoplankton can provide corals with an important source of nitrogen [28], [72]. At Ningaloo Reef, picoplankton dominates the phytoplankton community virtually year-round and picoplankton have been suggested to be an important nitrogen source for the coral community [17], [18]. Picoplankton is also possibly important for the maintenance of storage lipids. Our data suggests heterotrophy could be more important than autotrophy in supporting lipid synthesis as also previously shown for other coral species [73], [74]. Our results further suggest that heterotrophic feeding on picoplankton, in particularly picoeukaryotes, is also important for the maintenance of healthy zooxanthellae density and chl *a* concentrations in coral tissues. This has previously been observed and was explained by an increased supply of nitrogen from the coral host to the symbiont resulting in increased symbiont growth [75], [76].

The negative correlation between protein concentration and lipid ratio with chl *a* in the size fraction 2–5 µm and picoplankton could be the result of a time-lag effect, since an increase in protein and lipid concentrations with increased food availability can only be detected after weeks to months with increased food supply [20], [36], [77].

#### A. spicifera

Results of this study revealed a different pattern for *A. spicifera* with nitrogen, temperature and zooplankton as the dominant predictors of the measured health indices. A previous study showed that inorganic (75%) and organic (24%) nitrogen sources can provide up to 100% of the nitrogen required for tissue growth [35]. Thus results imply that both NH_4_ and other components of TN could be an important nitrogen source for *A. spicifera* at Ningaloo Reef.

Light was not the main predictor for any of the measured health indices in *A. spicifera*, suggesting that this species is less dependent on photoautotrophy than *A.digitifera* and possibly gains less carbon-rich products through photosynthesis. Corals' uptake of NH_4_ is light dependent [35] since synthesis of NH_4_ into amino acid is only possible if enough carbon, mainly supplied through photosynthesis is available [22]. The positive correlation between coral protein concentration and zooplankton carbon suggests that zooplankton might be an important source for daily carbon requirements in protein synthesis [19], [78]. We hypothesized that protein synthesis increases with an increase in current speed; increasing currents can reduce boundary layer thickness around corals and thus increase uptake rates of nutrients such as NH_4_ [41], [79]. Under optimum metabolic conditions, we note that protein concentrations were in fact inversely correlated with current speed. This suggests a more complex pattern of dependence between current speed and particle uptake, possibly involving the dependence on the physical mechanics of settling particles, for example. While of interest, this effect is beyond the scope of the current study.

Given that it takes weeks to months to detect a change in protein concentrations with changes in food availability [20], [36], [77], it was surprising that NH_4_ concentration as well as zooplankton showed a positive correlation with protein concentration for *A. spicifera*. Our previous study showed high metabolic rates (protein concentrations and RNA/DNA ratios) for *A. spicifera* [42] and we hypothesise that protein synthesis can react within hours/days to available food sources in the water. In further support of this hypothesis, temperature was positively correlated with protein concentratition and RNA/DNA ratio suggesting that metabolic rates increase with increasing temperatures as shown previously [80].

Previous studies have shown changes in zooxanthellae density and coral tissue chl *a* to be correlated with NH_4_ in the surrounding water [61], [72]. In our study, however, the negative correlations between NH_4_ and zooxanthellae density, and between TN and chl *a* concentration in the coral tissue, may be due to a time lag effect, since changes in zooxanthellae density are detectable only after weeks with increased NH_4_ supply [77], [81]. The positive correlation of picoeukaryotes and zooplankton with chl *a* concentration in the coral tissue might be explained by an additional shading effect, since high plankton concentrations result in low PAR levels in the water column [82].

#### Comparison between species

We hypothesise that the dependence of health indices of *A. digitifera* and *A. spicifera* on different physico-chemical factors might be at least partly related to differences in coral morphology. Coral shape has been shown to directly affect flow patterns, turbulence and the thickness of the boundary layer around corals, and thus impacts uptake rates of particulate and dissolved nutrients [41], [79]. Branching species rely at least to some extent on particulate matter [83] and have been shown to be better adapted to high light intensity [84], [85] while plate-shaped corals encounter higher rates of fine suspended particles [86]. These differences might partly explain why variations in RNA/DNA ratio and chl *a* concentration in the tissue of the caespito-corymbose shaped *A. digitifera* are better explained by PAR and particulate feeding while variations in protein concentration, lipid ratio and autotrophic indices in plate-shaped *A. spicifera* are better explained by TN and NH_4_ in the water as well as current speed. In short, *A.spicifera* seems more dependent on heterotrophy than the branch-like *A.digitifera.*

*A. digitifera* builds up and stores higher concentrations of lipids than *A. spicifera* [42] and might therefore rely more strongly on autotrophy than *A. spicifera* to fulfil their energy needs. This is in agreement with previous studies, which have shown heterotrophic feeding to support lipid synthesis [19], [70], [71]. Since the results presented here are based only on data collected during daylight hours, further data is required for the importance of nocturnal food sources for coral metabolism. It is assumed that corals feed primarily during night, when zooplankton densities are highest [87], [88]. We address this elsewhere (unpublished data). Since the physico-chemical factors we measured did not explain all of the variability in health indices, other factors such as bacterial uptake [17], [89] or nitrogen fixation through coral-associated bacteria [90] could be also important.

### La Niña and its impacts on physico-chemical factors and thus coral health

#### Differences in physico-chemical factors between La Niña and normal years

Light, current speed, temperature, nutrients and plankton concentrations off Ningaloo Reef in summer 2011 (February) differed from average values of previous years (1982–2008) [14], [17], [43] with temperatures more than 2 degree higher [46], lower PAR levels (around 30 instead of 55 mol photons m^−2^ d^−1^) [14] and higher current speed for summer than winter, which is normally the period of highest wave height at Ningaloo Reef [91]. NO_x_ and NH_4_ concentrations in February 2011 were also more typical of the higher concentrations normally observed in autumn, and chl *a* concentration in the water exceeded typical winter values [14]. These differences were a result of the strongest La Niña event on record, an anomalously high air-sea heat flux into the ocean and a record strength Leeuwin Current [46], which delivered high nutrient concentrations, and as a consequence, a phytoplankton bloom, high chl *a* and picoplankton concentrations as well as high zooplankton concentrations. The shift from *Prochlorochoccus* to *Synechoccocus* and picoeukaryotes reflect a clear nutrient input to the system [92].

#### Physico-chemical predictors for coral health during La Niña event

During the La Niña event, increases in plankton gained importance as a predictor for health indices of *A. digitifera* and *A. spicifera*, while temperature lost importance. This was unexpected since temperature has been shown to strongly affect autotrophic indicators as well as metabolic rates in corals [80], [93] and abnormally high temperatures during La Niña resulted in bleaching in some areas of Ningaloo Reef [46]. The significance of temperature may have been underestimated since the multivariate analysis was based on linear regression while temperature followed a quadratic function. In general, metabolic rates (measured as respiration rates) show an increase with temperature until a maximum is reached and decrease there after [80], [94]. In our study this trend was observed for protein concentrations and RNA/DNA ratio with temperature. Our results show that coral metabolism for both morphologies and in the case of *A. digitifera* also energy storage (as lipid ratio) was highest at 26 to 28°C (autumn) pointing towards optimal health at this temperature, in accordance with a previous study [95]. Zooxanthellae densities at these temperatures were lowest despite being within the range typical during normal conditions (ca. 1 to 2.5 × 10^6^ cm^2^) [96], [97]. This suggests that higher algal densities and chl *a* concentrations in the tissue together with low metabolic rates may reflect a decline in coral health.

An increase in autotrophic indices for abnormally high temperatures contradicted with results of previous studies, which show these indices to decline with increasing temperatures [64], [93] resulting in coral bleaching [2]. Low light levels together with a high supply of nitrogen might have counteracted the effect of high temperatures, since light has been shown as an important co-factor mitigating the coral bleaching response [98], [99]. High nitrogen concentrations result in the retention of photosynthate for the symbiont's own requirement and increased symbiont growth rates [26], [100]–[102]. This might explain the relatively high zooxanthellae densities and chl *a* concentration found in *A. digitifera* in February 2011, as well as picoeukaryotes and nitrogen (NH_4_ and TN) being the driving physico-chemical factors for autotrophic indices at this time. Given that low light conditions have the biggest impact on symbiont growth and lead to increased photoacclimatisation potential [36], [73] this could also explain the significant correlation between PAR and autotrophic indices during La Niña for *A. digitifera*.

Under the abnormal conditions during La Niña, our data support the hypothesis that less photosynthate is transferred to the coral host, and instead heterotrophically derived carbon and nitrogen is required to keep metabolic rates up [19], [30]. This was also reflected in correlations between plankton concentrations (picoplankton concentrations, chl *a* in the 2–5 µm size fraction as well as small zooplankton) and metabolic indices. Overall, our measured health indices showed that *A. digitifera* under stress conditions expresses lower metabolic indices and energy stores possibly due to a draw up of protein and lipid stores to sustain their metabolism [20], [30] since heterotrophic feeding seems not to provide sufficient carbon or nitrogen to the coral.

A. *spicifera* showed a different pattern to *A. digitifera*. Metabolic rates during the La Niña period were low [42] and PAR as well as phytoplankton (picoplankton and chl *a* in 2–5 µm fraction) concentrations were the main predictors for variations in metabolic indices. This suggests that *A. spicifera* relies more strongly on autotrophy. Since metabolic rates in *A. spicifera* depend strongly on temperatures overall, the abnormally high temperatures during La Niña are likely to strongly impact metabolic rates. Some coral species have shown a decline in protein corrections with abnormal temperatures [20], [30]. Increased zooxanthellae density in *A. spicifera* during La Niña was only correlated with PAR, suggesting that it is a pure photoacclimatisation response [60], [103] and not significantly related to nutrient supply through heterotrophic feeding. Overall our indices suggest that autotrophy combined with heterotrophy during stress events is not sufficient to supply enough energy to sustain high metabolic rates in *A. spicifera.*

### Implication for long-term health of Ningaloo Reef

Our study demonstrates a strong impact of extreme events on coral health. At the highest current speed, for example, coral cover was extremely low. Furthermore, the shift in the main physico-chemical predictors for coral health during the La Nina event indicates that during such an event, corals depend more heavily on resource availability. However, species' food preference also seems to play a role: though zooplankton and nutrient concentrations in the water were high during La Niña, the general preference was towards picoplankton, possibly due to their importance as a nitrogen source and their presence in relatively high concentrations year-round [17], [28], [69]. While high sea surface temperatures did not result in bleaching of corals, high seawater temperature reduced coral metabolism and lipid stores and hence, coral health. In analysing this process, we show that high chl *a* and zooxanthellae densities are not an appropriate indicator for good coral health, thus we recommend the use of metabolic indices and energy stores instead of autotrophic indices when assessing the health of natural coral populations.

Overall our results imply that changes in hydrodynamic regimes, temperature, light and availability of particulate and dissolved nutrients all impact coral health. However, it is the very specific combination of factors and sensitivities that determines the actual outcome for a given coral community, such that no single index or environmental factor can be seen to dominate. It should be noted that there are differences in biogeochemical responses to short term extreme temperature and long term temperature rise. Climate change and human impacts will impact coral health at Ningaloo reef, and are likely to impact coral community composition. However, when predicting the outcome of climate change, we suggest very emphatically that the devil is in the detail.
